# Gastroenteritis Outbreak Associated with Unpasteurized Tempeh, North Carolina, USA

**DOI:** 10.3201/eid1909.130334

**Published:** 2013-09

**Authors:** Stephanie E. Griese, Aaron T. Fleischauer, Jennifer K. MacFarquhar, Zackary Moore, Cris Harrelson, Anita Valiani, Sue Ellen Morrison, David Sweat, Jean-Marie Maillard, Denise Griffin, Debra Springer, Matthew Mikoleit, Anna E. Newton, Brendan Jackson, Thai-An Nguyen, Stacey Bosch, Megan Davies

**Affiliations:** Centers for Disease Control and Prevention, Atlanta, Georgia, USA (S.E. Griese, A.T. Fleischauer, J.K. MacFarquhar, M. Mikoleit, A.E. Newton, B. Jackson, T.-A. Nguyen, S. Bosch);; North Carolina Division of Public Health, Raleigh, North Carolina, USA (S.E. Griese, A.T. Fleischauer, J.K. MacFarquhar, Z. Moore, C. Harrelson, A. Valiani, D. Sweat, J.-M. Maillard, D. Griffin, D. Springer, M. Davies);; Buncombe County Department of Health, Asheville, North Carolina, USA (S.E. Morrison)

**Keywords:** Salmonella enterica serovar Paratyphi B, foodborne illness, soy foods, food handling, tempeh, North Carolina, salmonellosis, enteric infections, gastroenteritis, Salmonella spp., bacteria

## Abstract

During an investigation of an outbreak of gastroenteritis caused by *Salmonella enterica* serovar Paratyphi B variant L(+) tartrate(+), we identified unpasteurized tempeh as a novel food vehicle and *Rhizopus* spp. starter culture as the source of the contamination. Safe handling of uncooked, unpasteurized tempeh should be emphasized for prevention of foodborne illnesses.

Infections with *Salmonella* spp., a leading cause of hospitalizations and death among persons with foodborne illness in the United States, are most often associated with contaminated poultry or eggs (*1**,**2*). *S. enterica* serovar Paratyphi B variant L(+) tartrate(+) (formerly *Salmonella* var. Java) accounted for 1.1% of *Salmonella* infections reported to the Centers for Disease Control and Prevention (CDC) in 2009 (*3*). We investigated an outbreak of gastroenteritis caused by *S. enterica* ser. Paratyphi B var. L(+) tartrate(+) in North Carolina, USA, and found that the infections were associated with contaminated *Rhizopus* spp. starter culture and unpasteurized tempeh, a meat substitute, as a novel food vehicle.

## The Study

On March 30, 2012, a local health department notified the North Carolina Division of Public Health (NCDPH) of 5 persons who had laboratory-confirmed infection with *S. enterica* ser. Paratyphi B var. L(+) tartrate(+) and 3 epidemiologically linked persons who also had gastroenteritis. All 8 ill persons ate or worked at the same restaurant in Buncombe County, North Carolina; 5 (63%) were food handlers. Patient interviews did not identify a common source or vehicle for the infection. On April 24, NCDPH was notified of 10 additional persons with laboratory-confirmed *S. enterica* ser. Paratyphi B var. L(+) tartrate(+) infection; all had visited or resided in Buncombe County during the infection’s incubation period. 

Pulsed-field gel electrophoresis (PFGE) patterns of isolates from all 15 laboratory-confirmed case-patients were indistinguishable and represented a pattern not previously reported to the national database of enteric PFGE patterns, PulseNet, coordinated by CDC (www.cdc.gov/pulsenet; outbreak strain identification *Xba*1 JKXX01.1228). NCDPH initiated an investigation to determine the extent of the outbreak, identify the transmission source, and implement control measures.

A confirmed case was defined as laboratory identification of the outbreak strain from a person’s clinical specimen, regardless of illness onset date or exposure location. A probable case was defined as gastroenteritis in a person epidemiologically linked to a confirmed case. Passive reporting was enhanced through media reports and provider alerts. Active case finding was performed by hospital-based public health epidemiologists. Patients were interviewed by using the standard NCDPH salmonellosis reporting form to assess clinical symptoms; travel history; and food, water, and animal exposures.

**Figure 1:**
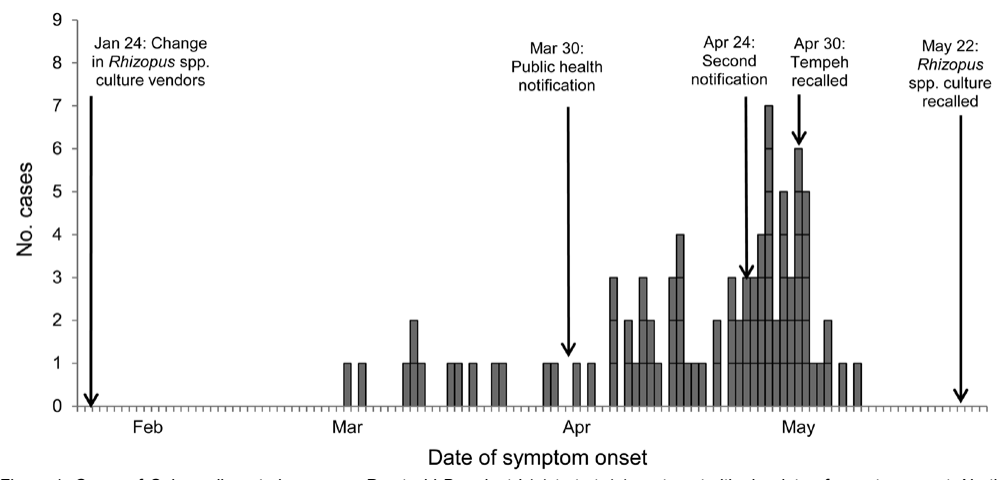
Cases of *Salmonella enterica* serovar Paratyphi B variant L(+) tartrate(+) gastroenteritis, by date of symptom onset, North Carolina, USA, February 29–May 8, 2012. For comparison, the date that the manufacturer of Brand A tempeh changed *Rhizopus* spp. starter culture vendors and dates of public notifications and recalls are indicated.

A total of 89 cases (87 confirmed, 2 probable) were identified among residents of 5 states; illness onset dates were February 29–May 8, 2012 (Figure 1). Of the 89 case-patients, 81 were residents of North Carolina; 80 reported travel to or residence in Buncombe County during the incubation period. Ten self-identified as food service workers, 2 as health care providers, and 39 as students of or visitors to University A, located in Buncombe County. All 86 patients for whom data were available experienced diarrhea (>3 loose stools in a 24-hour period; median duration 7 days, range 2–24); 30 (37%) of 82 reported bloody diarrhea (denominator reflects the number of case-patients who responded to the question). Eighty-three patients sought medical care; 8 were hospitalized; and none died (Table).

**Table:** Characteristics of 89 case-patients identified during investigation of infections with *Salmonella enterica* serovar Paratyphi B variant L(+) tartrate(+), North Carolina, USA, February 29–May 8, 2012*

Characteristic	No. (%) patients
Case classification	
Confirmed	87 (98)
Probable	2 (2)
Sex	
F	45 (51)
M	44 (49)
Patient age, y	
Range	4–79
Median	26
Mean	30
Signs or symptoms	
Diarrhea, n = 86	86 (100)
Abdominal cramps, n = 82	70 (85)
Fever, n = 84	69 (82)
Vomiting, n = 84	33 (39)
Bloody diarrhea, n = 82	30 (37)
Treatment and outcomes	
Sought medical care	83 (93)
Hospitalized	8 (12)
Died	0 (0)

*Values are no. (%) except as indicated. n values indicate number of case-patients who responded to question.

Consumption of vegetarian cuisine was commonly reported by case-patients. Because meat substitutes (e.g., tofu and tempeh) were not included on the standard reporting form, an outbreak-specific questionnaire was designed to gather more detailed exposure history. The first 50 patients identified received this questionnaire; 41 (82%) responded. Of these, 18 (44%) indicated that they had eaten tempeh, a fermented bean product that is usually pasteurized and cooked before consumption. Of these 18 patients, 12 had eaten tempeh at a restaurant, 4 had eaten it at University A, and 2 had sampled it at a grocery store. Two of the 18 persons who consumed tempeh also regularly handled it at a restaurant.

Site visits were conducted at the 3 restaurants most frequently identified in patient interviews (12%–40% of patient reports). Interviews with managerial staff and observation of food preparation identified multiple opportunities for cross-contamination, including preparation of uncooked, unpasteurized tempeh on the same surfaces used to prepare ready-to-eat (RTE) foods; failure to perform handwashing after handling uncooked tempeh; and bare-hand contact with RTE foods.

On April 26, the North Carolina Department of Agriculture and Consumer Services (NCDA&CS) notified NCDPH that salmonellae had been presumptively identified from samples of Brand A tempeh, which had been collected for routine food product testing before this outbreak was reported. Brand A produced unpasteurized tempeh in Buncombe County and distributed it to 34 restaurants in North Carolina. Additional distribution sites included grocery stores in several southeastern states and University A’s cafeteria. All 41 case-patients who completed the outbreak-specific questionnaire had eaten at a restaurant or venue that served Brand A tempeh. No cases were linked to grocery stores outside North Carolina.

NCDA&CS and NCDPH visited Brand A tempeh’s production facility to interview staff, review tempeh production, and obtain food samples. Of 6 employees, none reported recent illness or international travel. Production of the tempeh began in October 2009 and involved combining beans (e.g., soybeans, black beans, or black-eyed peas), vinegar, and *Rhizopus* spp. starter culture. The starter culture was added after cooking; the bean product was then fermented until it formed a dense cake, and the unpasteurized product was packaged, frozen, and shipped.

**Figure 2:**
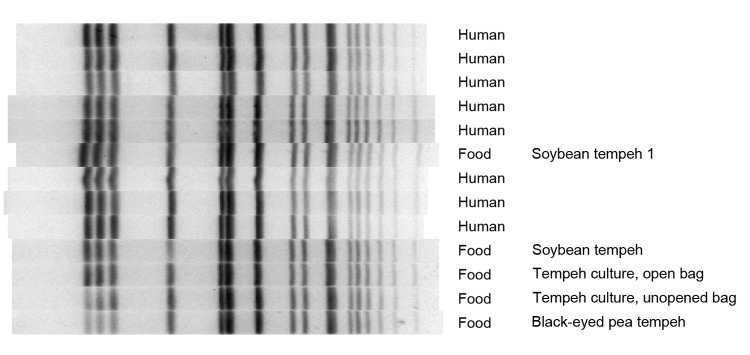
Pulse-field gel electrophoresis dendrogram showing *Xba1* enzyme band patterns for 8 case-patients, tempeh, and *Rhizopus* spp. starter culture associated with outbreak of *Salmonella enterica* serovar Paratyphi B variant L(+) tartrate(+) gastroenteritis, by date of symptom onset, North Carolina, USA, 2012.

Product testing revealed that Brand A tempeh was contaminated with *S. enterica* ser. Paratyphi B var. L(+) tartrate(+) that had a PFGE pattern matching the outbreak strain. *Salmonella* spp. were not recovered from raw soybeans or black-eyed peas, black beans were unavailable for sampling, and vinegar was not tested, but the outbreak strain was identified in opened and unopened bags of *Rhizopus* spp. starter culture (Figure 2).

The makers of Brand A tempeh switched *Rhizopus* spp. vendors in January 2012 and began using the new culture on January 24. The new *Rhizopus* spp. culture was produced in Indonesia and distributed internationally. Brand A tempeh was recalled voluntarily on April 30, 2012 (*4*). The *Rhizopus* spp. culture was recalled voluntarily, domestically and internationally, on May 22, 2012 (*5*).

## Conclusions

An outbreak of 89 cases of gastroenteritis related to infection with *S. enterica* ser. Paratyphi B var. L(+) tartrate(+) occurred in North Carolina during February–May 2012. The outbreak source was a *Rhizopus* spp. culture used in Brand A tempeh, which then acted as a novel vehicle for spreading salmonellae to consumers, probably through cross-contamination of RTE foods.

NCDPH confirmed the association between illness and Brand A tempeh through patient interviews and laboratory testing. The contaminated starter culture was distributed internationally; it is unclear why cases related to other tempeh brands did not occur, but a hypothesis is that, unlike other commercial tempeh products, Brand A tempeh is unpasteurized, and thus pathogens remained in the finished product.

The role of cross-contamination in foodborne outbreaks is well established (*6**–**11*). Bacteria can be transferred from surfaces to food products hours after surface contamination (*6**,**7**,**9*). RTE foods typically do not include a heating or cooking step to kill pathogens; consequently, raw vegetables and salads are commonly associated with foodborne outbreaks caused by cross-contamination (*7**,**10**–**12*). In this outbreak, all case-patients who responded to an outbreak-specific questionnaire reported eating at a venue that served Brand A tempeh. Although fewer than half recalled eating or handling tempeh, other case-patients might have been exposed during handling or consumption of cross-contaminated RTE foods.

Control measures addressing bare-hand contact with RTE foods, sanitation of food contact surfaces, and separation of raw and RTE foods were provided to restaurants that received Brand A tempeh and to the local Independent Restaurant Association. Correct handling of raw, unpasteurized tempeh was emphasized. Although tempeh can be part of a healthy diet (*13*), public health considerations should focus on safe handling of unpasteurized tempeh to prevent illness.
